# Inactivated vaccine against Aujeszky’s disease

**DOI:** 10.14202/vetworld.2021.2957-2963

**Published:** 2021-11-24

**Authors:** Zhanat B. Kondibaeva, Bolat A. Yespembetov, Khairulla B. Abeuov, Assiya K. Mussayeva, Sarsenbek T. Siyabekov, Saltanat T. Nussupova, Elmira K. Akmatova, Yerlan K. Pazylov, Kydyrbay T. Maikhin, Nazym S. Syrym

**Affiliations:** 1 Laboratory of Diagnostic of the Infectious Diseases , Research Institute for Biological Safety Problems, Gwardeiski, Kazakhstan; 2 Laboratory of Microbiology , Research Institute for Biological Safety Problems, Gwardeiski, Kazakhstan; 3 Bacteriology Laboratory , Kazakh Scientific Research Veterinary Institute, Astana, Kazakhstan; 4 Department of Clinical Veterinary Medicine , Kazakh National Agrarian University, Almaty, Kazakhstan; 5 Laboratory o the diseases of domestic animals , Kyrgyz Research Institute of Veterinary named after A. Duysheev, Bishkek, Kyrgyzstan; 6 Laboratory of Diagnosis of Infectious Diseases, National Reference Veterinary Center Almaty Branch, Almaty, Kazakhstan

**Keywords:** adjuvant, Aujeszky’s disease virus, colostral immunity, immunogenicity, strain “Kordai.”

## Abstract

**Background and Aim:**

The Aujeszky’s disease, also known as Pseudorabies, remains one of the most problematic fulminant diseases in domestic animals, affecting the central nervous system. The study aimed to investigate the effect of an inactivated vaccine against Aujeszky’s disease based on “Kordai” virus strain.

**Materials and Methods:**

To test the inactivation of the “Kordai” strain (grown by the roller method in VNK-21/13 cell culture with an infectious titer of at least 7.5 lg TCD_50_/ml) which is causative of Aujeszky’s disease, next-generation teotropin and propolis preparations were usedin concentrations of 0.1%, 0.08%, and 0.04%.

**Results:**

As a result of comparative studies on the optimization of parameters for inactivating the “Kordai” virus strain, it was established that teotropin is a more effective inactivant than propolis. At the same time, the optimal final concentration of teotropin for inactivation was 0.1%, along with a reaction medium temperature of 37°C, pH of 7.4-7.6, and duration of inactivation of 14 h. The titer of virus-neutralizing activity (VNA) of antibodies at the pH (neutralization reactions) in vaccinated sheep of 10-12 months of age was 7.5±0.3, Ig TCID_50_/ml (tissue culture infectious dose 50%), and 3.5±0.3 in the cell culture VNK-21/13 (culture of Syrian hamster kidney cells).

**Conclusion:**

To determine colostral immunity in newborn lambs, the method of metabolic status correction was used to vaccinate lambs obtained from immune sheep 4 months after birth. The results showed that lambs obtained from immune sheep had high VNA titers. A sustained immune response in vaccinated animals was obtained after double vaccination.

## Introduction

Aujeszky’s disease is an acute viral disease involving epizootic and sporadic cases. The disease is particularly recorded among pigs, dogs, and cats. The causative agent of Aujeszky’s disease is*Alphaherpesvirinae suid herpesvirus*, a species of viruses belonging to the genus *Varicellovirus*, family *Herpesviridae* [1]. Infection is usually derived from sick animals and virus carriers. In animals, alimentary involvement is predominantly found. According to the International Epizootic Bureau, Aujeszky’s disease is the most economically and socially significant epizootic disease. The last recorded outbreaks of this disease were in 2014 (in Romania), 2017 (in Papua New Guinea and Ukraine), and 2018 (in France) [2-6].

In recent studies on Aujeszky’s disease, efforts have been made to find new forms of vaccines that can induce earlier (colostral) immunity in vaccinated animals. Colostral immunity is a form of immunity that develops in newborns due to colostral immunoglobulins during the first 24-36 h of life. The creation of early post-vaccinal immunity primarily depends on the immunobiological reactivity of the animal, as well as the quantitative and qualitative characteristics of antigenic stimulation. Ultimately, it is necessary to develop vaccines that can stop the development of infection at an earlier stage [7-12]. The effectiveness of vaccines that cause a persistent immune response is associated with the following factors: (1) the quality and quantity of antigens; and (2) the choice of inactivants and adjuvants capable of enhancing the immunization process.

Although they are widely used to inactivate viruses, formaldehyde and ethyleneimine have adverse effects such as increased toxicity, reactogenicity, and immunosuppression. To overcome these, it is necessary to neutralize formalin, which increases the cost of the vaccine and, at the same time, complicates the manufacturing process. At present, there is particular interest in modern and harmless virus-inactivating agents such as teotropin and propolis. This work is a continuation of research aimed at increasing the immunogenicity of such vaccines that depend on selected inactivants [13,14] and adjuvants. The technology proposed in this paper differs in terms of its versatility, and the use of new adjuvants and inactivants compared with previously developed inactivated vaccines. The aim of this study was to develop an inactivated vaccine based on the “Kordai” virus strain.

## Materials and Methods

### Ethical approval

The conduct of animal experiments in scientific experiments during the implementation of this project was regulated by the “Code of Ethics” (1985), which includes the section “International recommendations for conducting biomedical research using animals,” and the Declaration of Helsinki of the World Medical Association (2000). All studies related to the use of animals were performed after receiving a positive conclusion from the local bioethical commission of the institute.

### Study period and location

The study was conducted from January to December 2019. The study was conducted at the Research Institute for the Problems of BioIgical Safety, Republic of Kazakhstan.

### Materials

It used a strain of Aujeszky’s “Kordai” disease virus, grown by the roller method in VNK-21/13 cell culture with an infectious titer of at least 7.5 Ig TCD_50_/ml. To inactivate vaccine strains, the inactivants teotropin and propolis were used. To test the parameters associated with inactivation of the “Kordai” viral strain causative of Aujeszky’s disease, next-generation teotropin and propolis preparations were used at concentrations of 0.1%, 0.08%, and 0.04%. In animals, Bartha K61 (e.g., Ingelvac®, Boehringer Ingelheim Vetmedica, USA Aujeszky MLV, an antigenic component labeled for the glycoprotein (gE)-attenuated Bartha K-61 strain of Aujeszky’s disease virus) induces an immune response to Aujeszky’s disease due to the causative virus 2-3 weeks after application. The effect lasts at least 4-6 months. The brood from vaccinated animals exhibits colostral immunity during the first 7-10 weeks of life. The vaccine is harmless and has no medicinal qualities. Competitive enzyme-linked immunosorbent assay for detecting anti-gE antibodies to Aujeszky’s disease virus is quite popular because it provides a result within 90 min and enables the differentiation of infected animals and those vaccinated with Bartha K-61. On the other hand, the “Kordai” inactivated vaccine does not contain DIVA qualities, but such an inactivated whole-virus vaccine allows the better isolation of gE glycoproteins (proteins that cause greater virulence of the wild-type virus than the isolated ADV strains). The optimal final concentration of the inactivant occurred at a temperature of 37°C, which corresponds to the reaction medium in which the virus was inactivated.

### Methods

The study investigated the properties of adjuvants, including aluminum hydroxides with saponin and Montanide Gel 01 (Seppic, France), peptide adjuvant V+XC55 (Russia), and chitosan.

The immunogenicity of vaccines was determined by a quantitative method on sheep of 10-12 months of age. For this purpose, three groups were used with eight animals in each, which were divided into four subgroups of two animals each. Dilutions of vaccines were prepared on “Placebo” according to the type of vaccine. Animals were immunized subcutaneously with the vaccine at dilutions of 1:3, 1:9, and 1:27 at a dose of 2 ml. Twenty-one days after vaccination, all vaccinated animals and two control ones were infected with the virulent virus of Aujeszky’s disease of the “Kordai” strain at a dose of 10^4^ TCID_50_/ml. The animals were clinically monitored for 14 days after the challenge. To study the humoral immunity in animals 10, 14, and 21 days after immunization, blood samples were taken for a neutralization (pH) study to determine the titer of neutralizing antibodies to Aujeszky’s disease.

To determine the avirulence, safety, and immunogenicity of the experimental vaccine series, sheep were used at 10-12 months of age, with a live weight of 30-35 kg. Blood samples were taken from lambs from immune sheep 2, 10, 14, and 21 days after birth, and then monthly. Serum was investigated in the neutralization reaction following the standard technique. The neutralizing activity of blood sera was determined using the neutralization index, which was calculated by the difference of logarithmic parameters between the normal and test sera. The calculation of 50% of the immunizing dose (ImD_50_) was carried out according to the Kerber-Ashmarin formula.

ImD50 = IgDn-IgG (∑ - I - where: 0.5),

IgDn-maximum dose tested;

IgG is the logarithm of the ratio of doses of vaccines;

∑ – sum of values;

I – number of surviving animals;

N – is the number of animals in the experiment.

ImD_50_ vaccine was 0.052 ml for cattle, sheep, and pigs and 0.021 ml for puppies. In terms of the volume of vaccine, it was more than 38 ImD_50_ for cattle, sheep, and pigs and 47 ImD_50_ for puppies.

## Results and Discussion

### Comparative study of inactivation of the virus causing Aujeszky’s disease by teotropin and propolis

**Figure-1:**
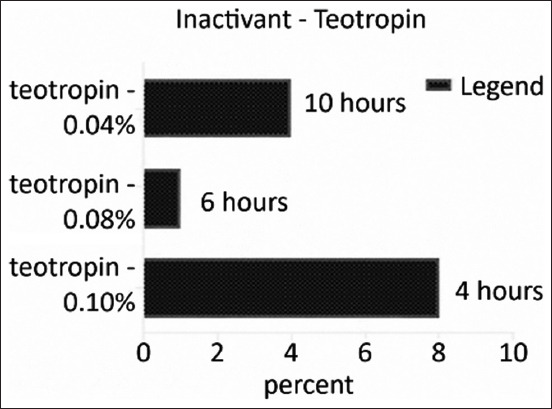
Results of the kinetics of inactivation of the strain “Kordai” of the Aujeszky virus by different concentrations of teotropin at a temperature of the reaction medium.

To test the parameters associated with inactivation of the Aujeszky’s disease-causing strain “Kordai,” next-generation teotropin and propolis preparations were used at concentrations of 0.08%, 0.04%, and 0.1%. The optimal final concentration of inactivant and the duration of virus inactivation at a temperature of the reaction medium of 37°C were determined. Figure-1 shows that, when using teotropin at concentrations of 0.1%, 0.08%, and 0.04%, the complete loss of infectious activity of the virus at the reaction medium temperature of 37°C occurred for 4, 6, and 10 h, respectively. The direct dependence of the duration of virus inactivation on the concentration of the inactivant in the reaction mixture was noted.

**Figure-2:**
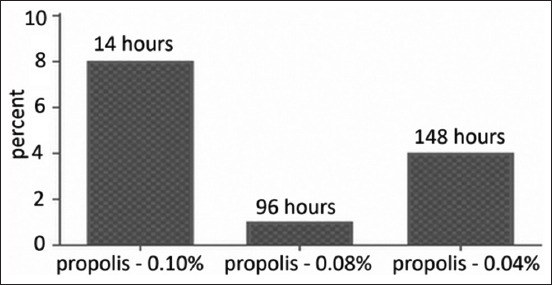
Results of inactivation of the strain “Kordai” of the Aujeszky virus by different concentrations of propolis at a temperature of the reaction medium 37°C.

**Figure-3:**
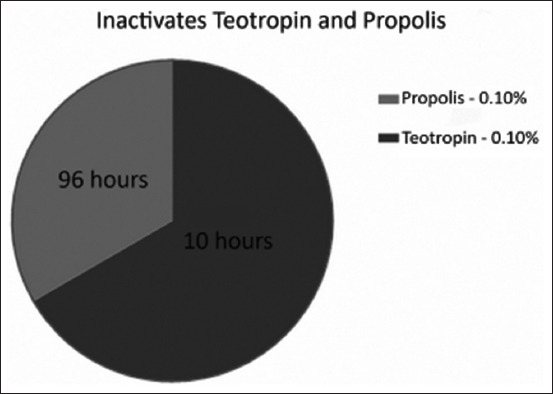
Results of chemical reaction at concentrations of 0.1% inactivants and reaction medium temperature 37°C.

As shown in Figure-2, the complete loss of infectious activity of the virus when using propolis at final concentrations of 0.1%, 0.08%, and 0.04% occurred at 14, 96, and 168 h, respectively. The next series of experiments involved comparative evaluation of inactivation of the virus causative of Aujeszky’s disease by teotropin and propolis. Both inactivants were used at a concentration of 0.1%. The results of inactivation of the virus causative of Aujeszky’s disease by teotropin and propolis at various concentrations are presented in Figure-3.

When using propolis, the inactivation time of the virus was markedly decreased (from 96 to 14 hours), with an increase in the concentration of inactivant and the temperature of the reaction medium. At a propolis concentration of 0.1% and a temperature of 37°C, the infectious properties of the virus causative of Aujeszky’s disease completely disappeared after 96 h; however, preparations made from a virus inactivated by this concentration of reagent did not possess immunogenicity. It was concluded that this inactivant is less effective for preparing an inactivated vaccine against Aujeszky’s disease.

To inactivate the strain “Kordai” causative of Aujeszky’s disease, the optimal parameters are as follows: Final concentration of teotropin 0.1%, temperature of the reaction medium 37°C, pH 7.4-7.6, and duration of inactivation 10 h. The toxicity of the vaccine against Aujeszky’s disease was tested on five rabbits with a live weight of no <2 kg. The vaccine was administered subcutaneously at a dose of 2 ml in the inner thigh area. At the administration site of the vaccine, a dense infiltrate (1.0×1.0 cm) formed, which later gradually resolved.

There was also (allowed) a short-term increase in body temperature to 39.5-40.0°C in the period up to 72 h after vaccination. The animals were monitored for 12 days after immunization. The vaccine was considered harmless, and all vaccinated animals remained clinically healthy during the follow-up period (with the exception of a local reaction to the vaccine).

### Selection of adjuvants for inactivated vaccine against Aujeszky’s disease virus-neutralizing activity (VNA) titer level in animals vaccinated with adjuvant vaccine

**Figure-4:**
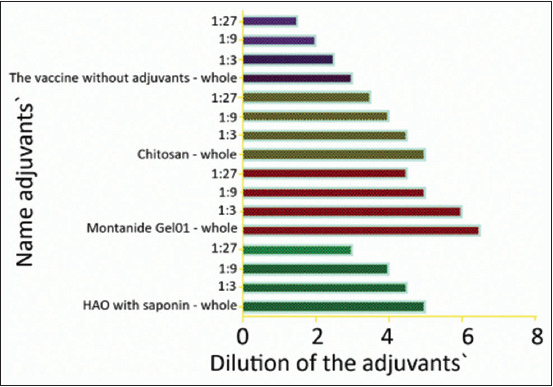
Virus-neutralizing activity titer level in animals vaccinated with an adjuvant vaccine.

Under experimental conditions in sheep, it has been established that manufactured vaccine samples based on different types of adjuvant have different antigenicity. The titers of VNA in sheep vaccinated with different variants of an inactivated vaccine against Aujeszky’s disease are presented in Table-1 and Figure-4.

**Table-1:** VNA titer level in animals vaccinated with adjuvant vaccine.

No.	Name of adjuvants	Dilution of the vaccine	VNA titer in log2 per day		

			10 days	14 days	21 days
1	GOA with saponin	Whole	5.0±0.2	5.5±0.3	6.0±0.4
		1:3	4.5±0.2	5.0±0.2	5.5±0.3
		1:9	4.0±0.1	4.5±0.2	5.0±0.2
		1:27	3.0±0.0	3.5±0.2	4.5±0.2
2	Adjuvants Montanide Gel01 (Seppic, France)	Whole	6.5±0.2	7.0±0.4	7.5±0.3
		1:3	6.0±0.1	6.5±0.2	7.0±0.3
		1:9	5.0±0.1	5.5±0.2	6.0±0.2
		1:27	4.5±0.1	5.0±0.2	5.5±0.2
3	Peptide adjuvants V+XC55 (Russia), or chitosan	Whole	5.0±0.2	5.5±0.1	6.0±0.3
		1:3	4.5±0.1	5.0±0.2	5.5±0.3
		1:9	4.0±0.1	4.5±0.1	5.0±0.2
		1:27	3.5±0.1	4.0±0.1	4.75±0.1
4	The vaccine without adjuvants	Whole	3.0±0.1	3.0±0.3	3.5±0.3
		1:3	2.5±0.1	2.75±0.1	3.5±0.3
		1:9	2.0±0.2	2.5±0.2	3.0±0.2
		1:27	1.5±0.1	1.5±0.2	2.0±0.2

The vaccine prepared with water-based adjuvant Montanide Gel 01 clearly had higher immunostimulatory activity than GOA with saponin and peptide adjuvant V+XC55. The titer of VNA antibodies at the pH in this group of animals was 7.5±0.3 TCID_50_/ml. This level was significantly different at p<0.05 compared with those in the other groups of animals. Vaccine samples with GOA adjuvants with saponin and V + XC55 peptide adjuvant showed a geometric mean titer of 6.0±0.4 Ig TCD_50_/ml. The vaccine without adjuvants showed an antibody titer of 3.5±0.3 TCID_50_/ml. The conducted experiments established that the most acceptable adjuvant in the inactivated vaccine against Aujeszky’s disease is Montanide Gel 01.

Montanide™ Gel is a range of ready-to-disperse innovative polymeric adjuvants designed to improve the safety and efficacy of aqueous vaccines. These adjuvants are based on the dispersion of highly stable gel particles of sodium polyacrylate in water. The depot effect with slow-release, due to polymer adsorption properties, improves the recruitment of the innate immune system. It provides significant enhancement of the immune response with a safety profile equivalent to that of aluminum salts.

### IMD_50_ doses of vaccine for animals diagnosed with Aujeszky’s disease

The levels of the immunizing dose (IMD_50_) of the vaccine against Aujeszky’s disease were studied in four groups of susceptible animals that had been vaccinated with the vaccine at dilutions of 1:3, 1:9, and 1:27. Fourteen days after vaccination, all vaccinated animals and a fifth control group were infected with the virulent Aujeszky’s disease virus at a dose of 10^4^ LDR (lethal dose rabbit) 50/ml for cattle, sheep, and pigs and 10^4^ LDR_50_/ml for puppies. Cattle, sheep, and puppies were immunized and infected subcutaneously, while pigs were vaccinated intramuscularly and infected subcutaneously. The animals were observed clinically for 21 days after infection. Surviving animals were considered to be immune. The results of the research are presented in Table-2.

**Table-2:** Determination of 50% of the immunizing dose of an inactivated vaccine against Aujeszky’s disease for various animal species.

Animal species	Grafting volume (ml)	Vaccine dilution			IMD50/ml	Amount of ImD_50_/ml vaccine volume

		1 : 3	1 : 9	1 : 27		
Cattle	2	2/2	2/2	2/2	0.052±0.2	38±0.4
Sheep	2	2/2	2/2	2/2	0.052±0.2	38±0.4
Porky	2	2/2	2/2	2/2	0.052±0.2	38±0.4
Puppies	1	2/2	2/2	2/2	0.021±0.3	47±0.2

n=3, p*<*0.05; confidence interval 0.95%. Notes: 1. Numerator – the number of protected animals. 2. The denominator is the number of animals in the experiment

The data in Table-2 show that none of the vaccinated animals reacted to the introduction of a virulent virus causative of Aujeszky’s disease. The general condition of the immunized animals during the entire observation period was satisfactory, and there were no clinical signs of the disease. In contrast, control animals died 5-11 days after infection.

Summarizing the above, the findings showed that a single vaccination of cattle, sheep, pigs, and puppies with vaccine against Aujeszky’s disease at dilutions of 1:3, 1:9, and 1:27 protected animals from infection with Aujeszky’s virus. Consequently, the vaccine with Montanide Gel 01 against Aujeszky’s disease contributes to the formation of robust immunity in vaccinated sheep and piglets for 4-6 days after vaccination.

In our opinion, by selecting the optimal concentration of Montanide Gel 01, it is possible to achieve maximum stimulation of the body’s immune system without increasing the reactogenicity of the vaccine. The data obtained are to some degree consistent with the results on the production of vaccines reported elsewhere, describing particularly high adjuvant activity of Montanide Gel 01 in antiviral vaccines [15-17]. However, no protection is absolute and immune responses vary between different individuals of the same vaccinated population [18]. Overall, the present study did not establish any relationship between different immune responses. The reason is that the study sample included healthy animals that did not respond or responded poorly to vaccination. After repeated injections, the proportion of non-immune animals decreased [19]. In addition, weight loss or gain is a less reliable criterion than VNA titers.

### Evaluation of colostral immunity to the virus causative of Aujeszky’s disease

**Figure-5:**
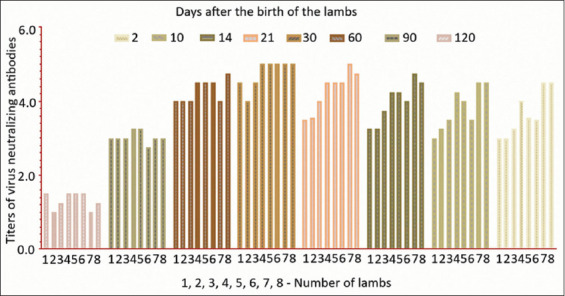
Dynamics of the formation of colostral immunity in lamb.

The next stage of our research was to determine the influence of colostral immunity on the antibody response of vaccinated lambs. For this, lambs obtained from immune sheep were vaccinated 4 months after birth. Here, the vaccine was administered subcutaneously at a dose of 1 ml at the inner side of the thigh (Table-3 and Figure-5).

**Table-3:** Dynamics of the formation of colostral immunity in lambs.

The number of animals	Days after the birth of lambs and VNA titer (log2)							

	2	10	14	21	30	60	90	120
1	3.0±0.19	3.0±0.19	3.25±0.19	3.50±0.08	4.5±0.12	4.0±0.07	3.0±0.19	1.5±0.14
2	3.0±0.25	3.25±0.19	3.25±0.14	3.56±0.09	4.0±0.25	4.0±0.25	3.0±0.09	1.0±0.14
3	3.25±0.19	3.5±0.14	3.75±0.19	4.0±0.12	4.50±0.14	4.0±0.12	3.0±0.12	1.25±0.19
4	4.0±0.19	4.25±0.12	4.25±0.12	4.5±0.04	5.0±0.08	4.5±0.19	3.25±0.24	1.5±0.14
5	3.56±0.14	4.0±0.19	4.25±0.19	4.5±0.14	5.0±0.12	4.5±0.21	3.25±0.12	1.5±0.12
6	3.5±0.19	3.5±0.08	4.0±0.14	4.5±0.12	5.0±0.19	4.5±0.12	2.75±0.24	1.5±0.12
7	4.5±0.12	4.5±0.1	4.75±0.12	5.0±0.25	5.0±0.9	4.0±0.14	3.0±0.12	1.0±0.19
8	4.5±0.14	4.5±0.12	4.5±0.25	4.75±0.19	5.0±0.12	4.75±0.19	3.0±0.12	1.25±0.12

Data from Figure-4 and Table-3 indicate that, after a single injection of the vaccine, it induced the formation of a high level of VNA in lambs against the background of colostral immunity. In the blood serum of lambs from immune sheep, there were high VNA titers, an increase of which was observed up to 21-30 days; then, they remained at the same level for a month and gradually decreased to 1.0-1.5 log2 by 4 months after birth. The study found an increase in the level of VNA to 4.0+4.5 log2 on 21 days after vaccination. Allergic reactions to the vaccine against Aujeszky’s disease in vaccinated lambsare not fixed, which indicates that this vaccine is safe to use.

Vaccine against the Kordai strain with the addition of the adjuvant Montanid Gel 01 was effective in lambs born from immunized sheep. Colostrum was rich in antibodies, indicating the effectiveness of preventive vaccination.

To prevent viral diseases, inactivated vaccines are widely used, which have a number of advantages over live vaccines. The term “inactivated” refers to the viability of the viruses that make up the vaccine. Adjuvants have the following requirements: They must be non-toxic at the doses used, should not cause adverse reactions in the body, should not possess antigenic activity, and should stimulate the development of long-term humoral and cellular immunity.

In recent years, there has been a renewed tendency to use inactivated vaccines. Inactivated vaccines are more stable and can be used for animals of any age and in reproductive herds. Such vaccines are used primarily for prophylactic purposes in safe farms and threatened areas. To test the inactivation of the “Kordai” strain of virus causative of Aujeszky’s disease, next-generation teotropin and propolis preparations were used at differentconcentrations. It was found that teotropin is a more effective inactivant than propolis. The optimal parameters for “Kordai” virus inactivation were found to be as follows: Final concentration of inactivant of 0.1% and reaction medium temperature of 37°C.

When testing various adjuvants based on an inactivated vaccine, it was found that, of three samples, the sample of the vaccine containing the adjuvant Montanide Gel 01 had the greatest immunostimulatory activity. A quantitative method was developed to control vaccine activity by determining the 50% immunization doses (ImD_50_), calculating the amount in one vaccination volume. Under the experimental conditions, ImD_50_ values for various types of susceptible animals, which provide a robust immune response after two immunizations, were determined. The findings showed that a comprehensive method to increase colostral immunity in newborn lambs had been developed using an approach of correcting metabolic status. An increase in the VNA level to 4.0±0.15 log2 for 21 days after vaccination was established. The formation of durable immunity in lambs containing maternal antibodies during the immunization period indicates the possibility of actively vaccinating newborns at 4 months of age.

## Conclusion

The vaccine against Aujeszky’s disease is non-toxic, sterile, has antigenic activity, and induces the formation of humoral immune response in animals. To test parameters affecting inactivation of the virus causative of Aujeszky’s disease, preparations of the next-generation teotropin and propolis were applied at concentrations of 0.1%, 0.08%, and 0.04%. The optimal parameters for virus inactivation with teotropin are as follows: Final concentration of inactivant of 0.1%, temperature of reaction medium of 37°C, and duration of inactivation 14 h. The avirulence of the viral suspension treated with 0.1% teotropin was confirmed by triple passaging of the material in VNK-21 cell culture and administration of the virus to rabbits. It was established that the created samples of vaccines based on different types of adjuvant possessed various antigenic activity. Immunizing agents were determined under experimental conditions for different types of susceptible animals, which provide a robust immune response after double immunization of animals.

## Data Availability

The datasets used and/or analyzed during the current study are available from the corresponding author on reasonable request.

## Authors’ Contributions

ZBK and YKP: Conceived and designed the analysis. BAY and AKM: Collected the data. STS and KTM: Contributed data or analysis tools. STN and KBA: Performed the analysis. EKA and NSS: Wrote the paper. All authors read and approved the final manuscript.
